# Analgesic efficacy of tapentadol in chronic joint disorders in horses: plasma serotonin concentration and adrenocortical response as biomarkers of pain-induced stress

**DOI:** 10.3389/fvets.2024.1505398

**Published:** 2024-12-17

**Authors:** Giovanna Lucrezia Costa, Marco Tabbì, Giuseppe Bruschetta, Filippo Spadola, Fabio Leonardi, Fabio Bruno, Nicola Maria Iannelli, Patrizia Licata, Francesco Macrì, Eraldo Sanna Passino, Daniele Macrì, Claudia Interlandi

**Affiliations:** ^1^Department of Veterinary Sciences, University of Messina, Messina, Italy; ^2^Department of Veterinary Sciences, University of Parma, Parma, Italy; ^3^Department of Veterinary Medicine, University of Sassari, Sassari, Italy; ^4^Zooprophylactic Institute, Palermo, Italy

**Keywords:** tapentadol, osteoarthritis, pain-induced stress, serotonin, cortisol, biomarkers, equine

## Abstract

The study aimed to evaluate the analgesic efficacy of tapentadol in horses, by determining plasma serotonin concentration and adrenocortical response, as biomarkers of pain stress in chronic joint disorders. Thirty-six horses (20 females and 16 males) were divided into three groups of 12 subjects each: group A, osteoarthritis (OA), grade 3–4 lameness; group B, OA, grade 5 lameness; and group C, no OA, no lameness, were enrolled. The orthopedic examination included flexion tests, and radiological and ultrasound examinations. The degree of lameness has been estimated from 0 to 5 according to the American Association of Equine Practitioners (AAEPs). Heart and respiratory rates (HR and RR) and blood pressure were recorded. Serotonin concentration and circulating cortisol levels were determined at baseline and the end of every week for 4 weeks. Biochemical parameters were recorded at baseline and the end of treatment with tapentadol. Subjects with OA were treated with tapentadol 0.5 mg kg^−1^. The response to painful stimulus on flexion tests was evaluated using the modified numeric pain rating scale (modified NRS 0–7) from baseline and the cumulative pain score (CPS 0–4) after the first week of treatment with tapentadol. The lameness decreased throughout the timeline in both groups (score from 3–4 to 1 in group A and score from 5 to 1 in group B) (*p* < 0.05). The NRS score decreased throughout the timeline (*p* < 0.05), from mild pain to no pain in group A (score 1–3 to 0) and from moderate pain to no pain in group B (score from 4 to 0). Physiological variables remained within the physiological range throughout the timeline. Cumulative pain scores ranged from 0.5 to 4 in group A and 1.5 to 7 in group B (*p* = 0.008). Serotonin concentrations remained unchanged throughout the timeline in all groups (*p* = 1.000) but in the OA groups, the concentrations were lower than control (*p* < 0.001). Circulating cortisol levels were reduced compared to baseline in subjects treated with tapentadol (*p* < 0.001). Tapentadol is effective in OA pain management in horses. Serotonin and cortisol may be utilized as biomarkers in the pain stress response. Serotonin can also determine the state of wellbeing of patients.

## 1 Introduction

Osteoarthritis (OA) is one of the most common and debilitating joint diseases in horses, responsible for 60% of cases of lameness and leading to significantly reduced athletic performance (1–4). This progressive, degenerative condition is characterized by the destruction of articular cartilage and chronic inflammation, which promotes disease progression and impairs joint function. OA also negatively impacts animal welfare, causing discomfort and pain (5–7).

Preventing the adverse consequences of acute pain sensitization during surgery and the development of chronic pain in horses with chronic joint diseases can significantly improve the animal’s overall wellbeing (8, 9). For pain management, multimodal analgesic therapies include a wide range of pharmacological agents, including non-steroidal anti-inflammatory drugs (NSAIDs), alpha-2 adrenergic agonists, N-methyl-D-aspartate antagonists (NMDAs), local anesthetic drugs, and opioids, such as tramadol and tapentadol (10–18). However, selecting an appropriate analgesic is essential to prevent peripheral and central sensitization following nociceptive stimulation, taking into account the etiology, severity, and chronicity of the inflammatory process (19–21). Opioids are effective in the management of mild to severe pain, both acute and chronic, due to their ability to act on G-protein-coupled opioid receptors located in the central and peripheral nervous systems (22, 23). The drug–receptor interaction results in neuronal hyperpolarization by interrupting the signal transmission to the higher nervous centers (24). The analgesic drugs, that activate the opioid receptors, particularly the μ-opioid receptor (MOR) subtype, have been used for decades to treat pain also in large animals (16, 25, 26). However, MOR agonists are not very effective in chronic conditions such as neuropathic pain. Many modes of action, including the activation of the descending noradrenergic pathway of pain inhibition, have been studied over the years since MOR stimulation causes both analgesia and adverse effects. Nevertheless, drugs that inhibit the reuptake of norepinephrine (NE) have been demonstrated to have an effective analgesic action, particularly in chronic pain (27).

Tapentadol (TAP) is an opioid drug that exerts dual central effects, acting as both a MOR agonist and a norepinephrine/serotonin reuptake inhibitor (NRI/SSRI) (28). The dual mechanism of action offers comparable analgesic efficacy to a molecule that solely acts as a MOR agonist while demonstrating a better profile with fewer side effects (29). Tapentadol is commercially available as a single-enantiomer molecule, the first member of a new pharmacological class of centrally acting analgesics termed MOR agonist noradrenergic reuptake inhibitors (MORNRIs) (30). It can be taken orally and is capable of blocking pain perception in the ascending pathways by exerting an agonistic effect on mu-opioid receptors (MORs) (31, 32). Compared to other opioids such as morphine, tapentadol exhibits a lower affinity for MOR receptors, approximately 50% less than morphine, but approximately double the affinity of tramadol. It has been shown to have adequate oral absorption and similar plasma bioavailability in dogs (for orthopedic pain) and rabbits (for surgical pain), as well as in human studies (for chronic pain) (33–35). However, there are no studies on the use of tapentadol in horses. The moderate affinity for MOR and the opioid-sparing effect of the NRI component means that TAP produces less intense and long-lasting side effects, with a 100-fold higher trigger threshold than classical MOR agonists. Preclinical pharmacological studies, *in vivo* and *in vitro,* have shown that TAP has weak anticholinergic activity and negligible inhibition of serotonin (5-HT) reuptake, but marked inhibition of NE reuptake (36, 37). This drug may represent a breakthrough in treating chronic pain in veterinary species, serving as an alternative to the opioid tramadol, which is commonly used for pain management in large animals (38, 39).

The use of tapentadol as a weak inhibitor of serotonin reuptake has been demonstrated to result in a reduction in adverse effects related to nausea and vomiting when compared to tramadol, which acts as a strong inhibitor of serotonin reuptake (40). Acute stress can induce increases or decreases in hippocampal and cortical 5-HT1A receptors, together with 5-HT2A, 5-HT1B, and 5-HT7, which are widely represented in leukocytes, and they are responsible for allowing the serotoninergic system to regulate cytokine production (41, 42). Acute stress, such as that induced by surgical procedures, is associated with a phenomenon known as stress-induced analgesia (SIA). The organism determines the instantaneous response, which increases the pain threshold significantly (43). In this physiological process, the endogenous opioid system (EOS) plays a fundamental role, working in conjunction with several other neurotransmitters and neuropeptides, including serotonin, noradrenaline, dopamine, gamma-aminobutyric acid (GABA), and many others to facilitate the process (44). This SIA condition is transient and is influenced by age, sex, and previous exposure to other stressful stimuli, even if the stress factor has been removed (45). In chronic conditions, post-trauma hypersensitivity may persist despite the absence of the stressor (46). In this context, cortisol, a steroid hormone synthesized from cholesterol, plays an essential role. It is released from the cortex of the adrenal glands into the bloodstream under the influence of ACTH (47). The secretion rates of cortisol are typically contingent upon several physiological factors, including breed, age, physical exercise, and circadian rhythm. The circadian rhythm of cortisol is subject to influence by a variety of factors, including the horse’s character, its diet, the training regimen, sleep patterns, lifestyle, and most significantly, the duration and frequency of acute or chronic stressors (48, 49).

Since the list of drugs approved for chronic pain management in horses is very limited, both in the EU and globally, it is important to promote effective analgesic practices for managing chronic pain in this species (50). The study aimed to evaluate the analgesic efficacy of tapentadol through the determination of plasma serotonin concentration and adrenocortical response as biomarkers to pain stress in chronic joint disorders in horses.

## 2 Materials and methods

The study was approved by the Review Board for Animals Care of the University of Parma (prot. No. 20/CESA/2023). The horses enrolled were not intended for food production or human consumption. The owners provided informed consent for the treatments administered to the horses. The drug administrations were recorded in the stable register.

### 2.1 Animals

Appropriate sample size was determined using Software G*Power 3.1. An “*a priori*” ANOVA (fixed-effects, omnibus, one-way) was conducted, with an effect size (*f*) of 0.50, a significance level of 0.05 (*α* err. Prob), a power of 0.70 (1-*β* err prob), and three groups. Thirty-six (20 females and 16 geldings) crossbred horses, aged 4 to 18 years and weighing approximately 400 kg, used for recreational purposes, were enrolled in this study (which lasted for a month in Sicily, from March to April, with temperatures ranging from 19 to 20°C). The horses were not used for any purposes other than the study. All the horses were from the same horse riding stable, kept under similar conditions, regularly vaccinated, and dewormed. The inclusion criterion was the presence of osteoarthritis (OA) or the absence of OA. The exclusion criterion was the possible administration of analgesic therapy.

### 2.2 Orthopedic examination and treatment

The horses underwent a clinical and orthopedic examination. After collecting the anamnestic data, the horse’s attitude while standing and moving (walk, small trot) was observed and the deformed joints were identified (presence of swelling). The deformed joints were palpated, and radiological, ultrasound, and joint centesis tests were performed to diagnose arthrosis (5). During the orthopedic examination, once the presence of lameness was ascertained, the degree was assessed by assigning scores ranging from 0 to 5 according to the American Association of Equine Practitioners (AAEPs). The points were assigned to the subjects by three independent observers, unaware of the pharmacological treatment and with no prior familiarity with the horses, with the following grading of clinical symptoms: a score of 0 was assigned when the lameness was not appreciable in any circumstances; score 1 was assigned for a grade of lameness difficult to observe in all circumstances; score 2 for lameness difficult to observe in specific condition as walking, trotting, weight carrying, circling, inclines, and hard surface; score 3 for lameness noticeable during trotting in any circumstances; score 4 marked lameness, nodding, hitching, and shortened strides; and score 5 lameness with minimal weight bearing in motion and inability to move (5). Horses were divided into the following groups: group A (*n* = 12 subjects) grade 3–4 lameness; group B (*n* = 12 subjects), grade 5 lameness; and group C (control group *n* = 12 subjects): no lameness. Horses in groups A and B orally received tapentadol (Palexia 50 mg, Grünenthal, Milan, Italy) at a dosage of 0.5 mg kg^−1^ for 4 weeks. The dosage of tapentadol used in horses was established by considering the dosage of tapentadol used in dogs for the management of chronic pain (10–30 mg kg^−1^) and the dosage of tramadol (0.2–3 mg kg^−1^) used in the management of acute pain in horses and cattle (17, 26, 39, 50). Additionally, the dosage was determined based on the assumption that horses with osteoarthritis showed moderate pain according to the NRS. Lameness scores were assigned at the time of the orthopedic examination and at the end of each week for 4 weeks after the tapentadol treatment.

### 2.3 Measurement of physiological parameters

Heart rate (HR), non-invasive systolic pressure (SAP), diastolic pressure (DAP), and mean arterial blood pressure (MAP), were measured by placing a cuff (12–19 cm in diameter) at the base of the tail, using a CAMS 2 multiparameter monitor (Forlì, Italy). Respiratory rate (RR) was detected by counting thoracic excursions over a 1-min period. Physiological parameters were detected and recorded, after 20 min of acclimatization in a special box, at baseline and at the end of each week for 4 weeks.

### 2.4 Measurement of hematological and biochemical parameters, serotonin concentration, and circulating cortisol levels

To determine the above parameters, a blood sample (20 mL) was taken from the jugular vein by the same operator. An aliquot (9 mL) was placed in a vacuum serum isolation tube (serum coagulation activator Z, VACUETTE®, Greiner Bio-One, Kremsmünster, Austria), cooled at 4°C, and centrifuged within 3 h at 3,000 *g* for 20 min to isolate the serum. Biochemical analysis was then conducted for the determination of the levels of glucose, aspartate transaminase (AST), alanine transaminase (ALT), blood urea nitrogen (BUN), and cortisolemia. Glucose was determined using the glucose oxidase/peroxidase method. AST and ALT levels were measured using kinetic methods at 37°C. These parameters were then quantified using a UV–Vis spectrophotometer (A560, Fulltech, Rome, Italy) at baseline and at the end of each week for 4 weeks after the treatment with tapentadol. Serum cortisol analysis was conducted in triplicate using an enzyme immunoassay (EIA, RADIM, Rome, Italy) and an automated analyzer (BRIO, SEAC, Rome, Italy). The samples were incubated with cortisol conjugated to horseradish peroxidase (HRP), competitive for the binding sites of the antiserum coating on the wells. After three washes, the chromogenic substrate (tetramethylbenzidine, TMB) was added, and after an incubation period of 30 min at 25°C, the optical density at 450 nm was measured using a spectrophotometer (Sirio S, SEAC, Florence, Italy) to evaluate the enzymatic activity (51). An aliquot of 6 mL was placed into an EDTA Vacutainer tube (K3-EDTA, VACUETTE, Greiner Bio-One, Kremsmünster, Austria). The platelet-poor plasma obtained by centrifugation at 4,500 *g* for 10 min at 4°C was used to determine 5-HT, through the use of an ELISA kit (BioVision Incorporated, Milpitas, CA, USA) (52). To each well, 50 μL of the standard or sample was mixed with 50 μL of the biotin-detection antibody working solution and incubated for 45 min at 37°C. After three washes, 0.1 mL of SABC working solution was added, and the plate was incubated at 37°C for 30 min. Following the washing process, 90 μL of TMB substrate was added, and the plate was incubated at 37°C for 30 min. Subsequently, the stop solution was added. The optical density values were subsequently read at 450 nm (A560, Fulltech, Rome, Italy). Serum cortisol and 5-HT were determined at baseline and at the end of each week for 4 weeks. Another aliquot of 1 mL of blood was placed into an EDTA Vacutainer tube to perform the complete blood count using IDEXX Italia (at baseline).

### 2.5 Response to painful stimulus and sedation assessment

The response to the painful stimulus was assessed by assigning scores, using a modified numeric pain rating scale (modified NRS), to the reactions evoked by the flexion test as follows: 0 = no pain, 1–3 = mild pain, 4 moderate pain, and 5–7 intense pain. Furthermore, the cumulative pain score (CPS) was evaluated after the first week of treatment, weekly, by assigning scores from 0 to 4 to the percentage changes in heart rate, respiratory rate, and systolic blood pressure, compared at baseline according to the following scheme: 0: =0%; 1: >0% but ≤10%; 2: >10% but ≤20%; 3: >20% but ≤30%; and 4: >30% (26). The sum of the three scores gave the total CPS, with a score of 10 serving as the cutoff point for determining whether the analgesic therapy was satisfactory. NRS scores were assigned without a baseline, while the cumulative pain score was performed after the first week of treatment with tapentadol. Sedation was assessed by calculating the percentage decrease in the chin’s distance from the ground in relation to its initial distance from the ground (HHAG), measured from the base of the chin to the ground using a measuring tape. The horse was placed on a horizontal surface for this evaluation. Ataxia was assessed by assigning numerical scores ranging from 0 to 3, as follows: point 0, no ataxia; point 1, the horse was stable, but it showed slight oscillations; point 2, the horse leaned on the handler; and point 3, the horse crossed its hind limbs. HHAG was evaluated weekly after the first week of treatment. All points were assigned by three independent observers unaware of the pharmacological treatment received by the horses (17).

### 2.6 Statistical analysis

Statistical analysis was performed using SPSS 27.1 (IBM Company, Novegro-Tregarezzo, Italy). The Shapiro–Wilk normality test was performed. The data were reported as median and range or mean and standard deviation (SD) as appropriate. Differences over time and among groups were assessed using the Wilcoxon signed-rank test followed by Mann–Whitney U-test and two-way ANOVA for repeated measures, followed by the Bonferroni test as appropriate. Inter-observer agreement was assessed using Kendall’s discordance coefficient (W). SPSS automatically corrects the data with the base 10 logarithm. Correlation between lameness scores and NRS was performed by calculating Pearson’s correlation coefficient. Statistical significance was set at a *p*-value of <0.05.

## 3 Results

The total sample size was 36 subjects with a sample actual power of 0.7. Inter-observer agreement was high (*W* = 1). The data were not normally distributed. All horses successfully completed the study. The degree of lameness decreased throughout the timeline in both groups (score from 3–4 to 1 in group A and score from 5 to 1 in group B) (*p* < 0.05). The comparison of the degree of lameness between the groups showed a statistically significant difference from baseline up to the second week of treatment with tapentadol between groups (*p* ≤ 0.005). In the third and fourth weeks of treatment with tapentadol, the lameness scores did not show statistically significant differences (*p* > 0.05) (Table 1). The NRS score significantly decreased throughout the timeline in both groups (score 1–3 to 0; *p* < 0.05) (Table 2). The correlation between lameness and NRS scores was not significant in both groups (*p* > 0.05). No ataxia was detected in any of the horses throughout the study. Physiological variables were within the normal range values for horses, throughout the timeline (Table 3). CPS scores ranged from 0 to 6 in group A and from 0 to 7 in group B (Table 4). Serotonin, cortisol concentration, and glycemia showed significant global differences at baseline among the groups (*p* = 0.000). Serotonin concentrations remained unchanged over time in all groups (*p* = 1.000), but the OA groups showed significantly lower serotonin concentrations than the control group (*p* < 0.001). The percent difference in serotonin concentration between groups A and B compared to group C was approximately 40–50% throughout the timeline (Table 5). Circulating cortisol concentrations in the OA groups were higher than in the control group (*p* < 0.001) and decreased throughout the timeline (*p* < 0.001). After the fourth week, there was no significant difference in circulating cortisol concentrations between groups OA and C (*p* > 0.05). The percent difference in circulating cortisol concentrations between groups A and B compared to group C is approximately 7–20% throughout the timeline (Table 5). Glycemia was higher in groups A and B than in group C at baseline (*p* < 0.001) and decreased throughout the timeline in both groups treated with tapentadol (*p* < 0.001). The percent difference in Glycemia among groups A and B vs. group C are of approximately 2/41% throughout the timeline (Table 5). Aspartate transaminase (AST), ALT, BUN, and blood count (data not showed) were in the physiological ranges for horses in the three groups and remained unchanged in the groups treated with tapentadol (Table 6).

**Table 1:** Lameness scores, in groups A and B assigned according to the American Association of Equine Practitioners (AAEPs), at baseline and at the end of each week for 4 weeks after the treatment with 0.5 mg kg^−1^ tapentadol.

	Baseline	Week			
		1	2	3	4
Group A	3 (3–4)^α^	1 (0–1)*^α^	0 (0–1)*^α^	0 (0–1)*	0 (0–1)*
Group B	5 (5–5)	3 (2–3)*	2 (1–2)*	1 (0–2)*	0 (0–1)*

The data are reported as median and (range); *difference throughout the timeline; ^α^difference between groups A and B; (the Wilcoxon signed-rank test followed by Mann–Whitney U-test).

**Table 2:** Response to the painful stimulus assigned according to a modified numeric pain rating scale (modified NRS), to the reactions evoked by the flexion test: 0 = no pain; 1–3 = mild pain; 4 = moderate pain, 5–7 = intense pain, at baseline and at the end of each week for 4 weeks after the treatment with 0.5 mg kg^−1^ tapentadol.

	Baseline	Week			
		1	2	3	4
Group A	2.5 (1–3)	1 (0–1)*	0 (0–1)*	0 (0–1)*	0 (0–0)*
Group B	1.5 (1–3)	0.5 (0–1)*	0 (0–0)*	0 (0–0)*	0 (0–0)*

The data are reported as median and (range); *difference throughout the timeline; (the Wilcoxon signed-rank test followed by the Mann–Whitney U-test).

**Table 3:** Physiological values for horses measured at baseline and at the end of each week for 4 weeks.

	Baseline	End of the week			
		1	2	3	4
HR (beats min^−1^)					
Group A	40 (38–42)	31 (28–34)*^α^	36 (30–40)	38 (36–42)	38 (36–40)
Group B	40 (38–40)	40 (38–42)	50 (50–52)*^α^	39 (38–40)	40 (38–42)
f_R_ (breaths min^−1^)					
Group A	16 (12–20)	10 (8–12)*^α^	16 (12–20)	16 (12–20)	16 (12–20)
Group B	20 (18–22)	23 (22–24)*	21 (20–22)*^α^	20 (18–20)	**20 (18–22)** ^α^
SAP (mmHg)					
Group A	97 (91–118)	97 (91–123)	93 (78–102)	99 (92–131)	102 (92–111)
Group B	**92 (91–96)**^α^	**92 (90–100)**^α^	104 (100–110)*^α^	84 (80–86)*^α^	84 (82–86)*^α^
MAP (mmHg)					
Group A	63 (55–73)	56 (49–81)	76 (58–85)*	64 (53–82)	66 (54–80)
Group B	66 (64–71)	58 (57–64)*	56 (53–62)*^α^	58 (52–64)*	56 (53–58)*
DAP (mmHg)					
Group A	44 (36–53)	57 (34–75)	50 (41–56)	48 (36–52)	42 (37–60)
Group B	56 (48–60)^α^	56 (53–58)	46 (44–48)*^α^	44 (37–50)*	44 (38–46)*

The data are reported as median and (range); *difference throughout the timeline; ^α^Bold indicates the difference between groups A and B; (the Wilcoxon signed-rank test followed by Mann–Whitney U-test).

**Table 4:** CPS measured at the end of each week for 4 weeks.

	Week			
	1	2	3	4
Group A	0.5 (0–1)	0.5 (0–2)	2.5 (1–4)	1.5 (0–6)
Group B	3 (1–4)^α^	6 (6–7)*^α^	0 (0–2)*^α^	0.5 (0–2)*

The data are reported as median and (range); *difference throughout the timeline; ^α^Bold indicates the difference between groups A and B; (the Wilcoxon signed-rank test followed by Mann–Whitney U-test).

**Table 5:** Serotonin, cortisol concentration, and glycemia measured at baseline and at the end of each week for 4 weeks.

	Baseline	End of the week			
		1	2	3	4
5-HT (ng/ml)					
Group A	23.55 ± 6.40^β^ **42.5%**	22.37 ± 4.4^β^ **48.4%**	22.62 ± 5.42^β^ **49.2%**	24.5 ± 3.27^β^ **36.3%**	24.28 ± 6.26^β^ **35.9%**
Group B	23.25 ± 3.35^γ^ **43.7%**	25.02 ± 6.21^γ^ **32.4%**	24.83 ± 3.13^γ^ **34.8%**	23.18 ± 2.4^γ^ **44%**	23.37 ± 2.04^γ^ **41%**
Group C	33.48 ± 2.13	33.1 ± 2.31	33.67 ± 2.17	33.38 ± 2.21	33 ± 2.20
Cortisol (μmol/L)					
Group A	184.98 ± 3.91^β^ **22%**	174.23 ± 4.19*^β^ **18%**	169.41 ± 3.84*^βγ^ **21.4%**	162.94 ± 3.37*^βγ^ **12.3%**	153.04 ± 4.04* 14%
Group B	186.47 ± 4.81^γ^ **22.7%**	175.15 ± 3.26*^γ^ **18%**	164.61 ± 2.59* 12%	159.91 ± 3.86*^γ^ **10%**	154.29 ± 4.26* 7%
Group C	143.13 ± 3.47	142.69 ± 4.73	144.79 ± 2.24	143.24 ± 2.96	143.97 ± 2.37
Glycemia (mg/dL)					
Group A	116.34 ± 3.26^β^ **41%**	110.24 ± 2.47^β^ **35%**	104.01 ± 3.18^βγ^ **32%**	86.16 ± 3.94* 31%	70.13 ± 4.61* 1.4%
Group B	121.81 ± 1.42^γ^ **44%**	115.49 ± 3.84^γ^ **43%**	108.67 ± 3.71^γ^ **38.7%**	89.43 ± 2.39* 35.7%	75.04 ± 3.91* 5%
Group C	68.21 ± 2.45	71.21 ± 2.37	69.54 ± 3.16	73.39 ± 3.16	71.11 ± 2.61

The data are reported as mean and SD; *difference throughout the timeline; ^β^difference between groups A and C; ^γ^difference between groups B and C; (ANOVA for repeated measures, followed by the Bonferroni test); Bold indicated the % percentage changes among groups A and B vs. C.

**Table 6:** Biochemical parameters measured at baseline and at the end of each week for 4 weeks.

Groups	Baseline	Week			
		1	2	3	4
AST (U/L)					
Group A	73.46 ± 2.41	74.24 ± 3.16	72.84 ± 4.21	74.36 ± 4.76	73.12 ± 4.57
Group B	72.20 ± 3.21	72.48 ± 62	73.01 ± 2.97	71.98 ± 3.34	74.21 ± 2.43
Group C	70.0 ± 2.71	71.06 ± 3.76	72.63 ± 3.52	71.94 ± 3.47	72.10 ± 3.14
ALT (U/L)					
Group A	8.82 ± 3.15	8.46 ± 3.74	8.57 ± 2.94	8.24 ± 3.14	7.67 ± 3.12
Group B	8.91 ± 3.61	8.64 ± 3.47	8.31 ± 3.66	8.01 ± 4.25	7.96 ± 3.95
Group C	8.19 ± 3.16	7.56 ± 3.77	8.06 ± 3.67	7.67 ± 3.91	7.34 ± 2.73
BUN (mg/dL)					
Group A	17.41 ± 3.14	17.56 ± 3.16	15.24 ± 2.93	14.26 ± 2.86	14.28 ± 2.28
Group B	19.45 ± 2.26	16.97 ± 3.39	16.37 ± 3.67	15.47 ± 3.29	14.66 ± 3.14
Group C	15.84 ± 4.27	14.61 ± 4.01	15.97 ± 3.74	13.49 ± 2.34	12.03 ± 2.87

Data are reported as mean and SD; *difference throughout the timeline; ^β^difference between groups A and C; ^γ^difference between groups B and C; (ANOVA for repeated measures, followed by the Bonferroni test).

## 4 Discussion

A tapentadol dose of 0.5 mg kg^−1^ is effective for the management of chronic OA pain in horses. In our study, plasma serotonin concentration did not change in OA subjects during the 4 weeks of tapentadol treatment. This may be due to the effective analgesic therapy, which improved the wellbeing of the treated patients. This may be supported by decreased cortisol levels and lower pain scores. Serotonin, like cortisol, may serve as a biomarker in the stress response associated with chronic pain (53). Many environmental, social, and clinical conditions can increase oxidative stress in animals, especially when painful pathologies occur. Therefore, OA-induced oxidative stress can cause an increase in serotonin and enzymes, such as superoxide dismutase (SOD), catalase (CAT), glutathione peroxidase (GPx), and glutathione reductase (GSR), which are involved in the antioxidant process (54). However, acute stress can induce an increase or decrease in serotonin concentrations (55). The neurotransmitter 5-HT plays a crucial role in various physiological and behavioral processes in all animals, including horses. It facilitates tissue regeneration, immune response, inflammatory modulation, and pain and stress management at the peripheral level (56).

The CPS scores remained below the established cutoff point, demonstrating good pain management with the oral administration of 0.5 mg kg^−1^ tapentadol. This result was also supported by the observation of physiological variables, which did not show an increase by 20% compared to baseline during 4 weeks of treatment. Similar results have been previously observed in cattle following the administration of tramadol at a dosage of 1 mg kg^−1^ during farriery and in cattle. Additionally, in the latter, three different dosages of tramadol were compared (1–1.5 to 3 mg kg^−1^) for analgesia and sedation, during transvaginal and transrectal ultrasound scans with biopsies (26, 39).

While it has been noted that the changes in physiological parameters remained within the normal range for horses, there appears to be a specific correlation between these events, especially regarding heart rate and respiratory rate. One-week delay between changes in these parameters was statistically significant when compared to their previous levels. This was especially noticeable in the group with higher lameness levels (group B), where significant statistical differences were seen after 1 week. This may be related to the dosage of tapentadol used because patients with a greater degree of lameness may have required a higher dosage of tapentadol as demonstrated in human and dog studies (33, 57). However, the NRS scores did not correlate with the degree of lameness. This should be addressed in future studies to establish a dosage appropriate to the actual needs of the patient. Regarding blood pressure, although the recorded values remained within the normal range, a decrease in blood pressure was observed in group B (the higher lameness group, but with a lower NRS score) after 3–4 weeks of treatment. The decrease in blood pressure could be related to the effects of the drug treatment. However, the two groups received the same dosage of tapentadol, and there was no correlation between the degree of lameness and the NRS scores. Furthermore, the blood pressure increased proportionally to the pain.

Serotonin concentrations remained unchanged over time in all groups even though groups A and B, suffering from chronic OA, showed significantly lower serotonin concentrations compared to the control group. These results could support the effectiveness of analgesic therapy with tapentadol. In physiological conditions, the plasma levels of 5-HT are usually lower than the increased concentration triggered by the inflammatory state (58). Peripheral 5-HT levels correlate with the development and resolution of immune- and inflammation-related diseases such as arthritis (59, 60). However, the role of 5-HT in OA and its changes in response to analgesic treatment remain to be clarified. High concentrations of 5-HT stabilize the hypothalamic–pituitary–adrenal axis response, whereas low concentrations facilitate aggressive or stress-related responses (61, 62). Serotonin levels differ considerably, from low concentrations observed in horses with critical pathologies and acute onset, to high concentrations measured in horses with chronic pathologies or subjected to surgery (63, 64). The serotoninergic system is activated by the stress stimuli through pre- and post-synaptic 5-HT receptors, with specific sensitivity to stressors (65). Circulating cortisol concentrations were reduced in the OA groups throughout the timeline, showing a reduction in pain-induced stress. It is known that any condition of stress and discomfort, including pain, induces the release of cortisol (53). Glycemia decreased during the tapentadol treatment period in horses with OA. Studies conducted in humans and dogs have demonstrated increases in glycemia after surgery and in horses during transport (53, 54). Biochemical parameters remained unchanged during treatment with tapentadol, which proved effective and safe in the management of chronic OA pain in horses.

The correlation between the lameness scores and NRS scores was not significant, probably due to the inadequacy of the scales used in the study, which allow only subjective assessments. This could represent a limitation of the study, which could be overcome by using appropriate scales with wider ranges or by increasing the number of subjects. Although the effect size (*f* = 0.50) and power (0.70) indicate a reasonable ability to detect medium-sized effects, further studies with a larger sample size or extended treatment duration may be warranted to confirm these findings. The observed reduction in cortisol suggests potential clinical efficacy for tapentadol as an analgesic for chronic osteoarthritis pain in horses. This, along with pain monitoring tools such as analog scales, supports the possible clinical application of tapentadol. Future research should address the persistence of analgesic effects after discontinuation of therapy and examine the pharmacokinetics of tapentadol in horses to optimize treatment strategies.

## 5 Conclusion

Serotonin concentrations in horses affected by OA showed no significant differences, while serum cortisol concentrations decreased compared to baseline, during therapy with tapentadol. This can support clinical monitoring with the use of analog scales, such as NRS, to demonstrate that tapentadol is a valid option in the treatment of chronic OA pain in horses.

## Data Availability

The original contributions presented in the study are included in the article/supplementary material, further inquiries can be directed to the corresponding authors.
